# Supplementary material for: Accurate and rapid antibiotic susceptibility testing using a machine learning-assisted nanomotion technology platform
Source: Nat Commun. 2024 Mar 18;15:2037. doi: 10.1038/s41467-024-46213-y (PMC10948838; doi:10.1038/s41467-024-46213-y)
Supplement: Supplementary file 1 — Supplementary Information [file 41467_2024_46213_MOESM1_ESM.pdf]

Supplementary Information

Content

**Supplementary Figures**	**2**
Supplementary Figure 1	2
Supplementary Figure 2	3
Supplementary Figure 3	4
Supplementary Figure 4	5
Supplementary Figure 5	6
Supplementary Figure 6	8
Supplementary Figure 7	10
Supplementary Figure 8	**11**
**Supplementary Text - Development of susceptibility and resistance classification models using advanced signal processing of nanomotions and machine learning techniques**	**12**
Methodology	12
Results	21

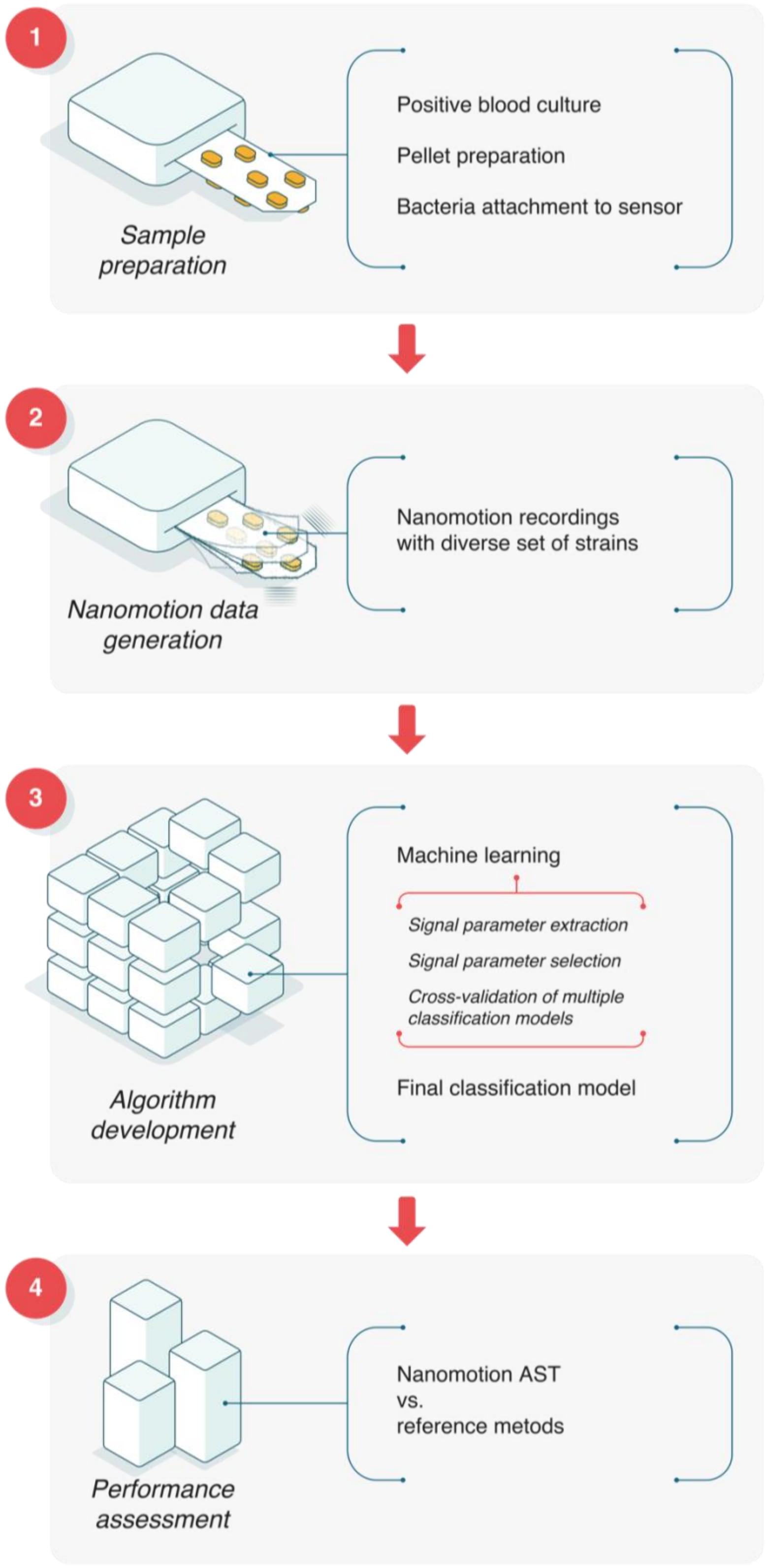

Supplementary Fig. 1 | Workflow of the nanomotion AST including the pellet preparation, nanomotion recording and machine learning to develop classification models that are benchmarked against reference AST methods.

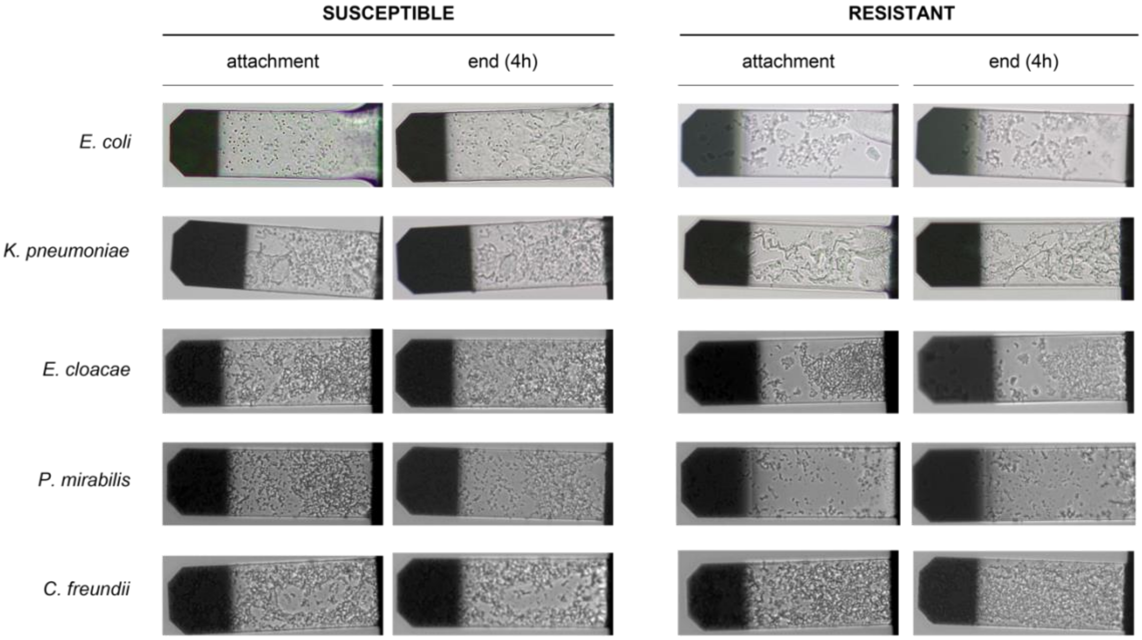

Supplementary Fig. 2 | Bacterial attachment to the cantilever using the bacterial attachment components shown in Figure 1c. Phase contrast images of cantilever attachments of several different *Enterobacteriaceae*. The cantilever dimensions of 130 µm × 40 µm × 0.75 µm. Each cantilever picture was taken immediately after attaching the bacteria (attachment) and after four hours (end) in 50% LB to mimic nanomotion AST conditions.

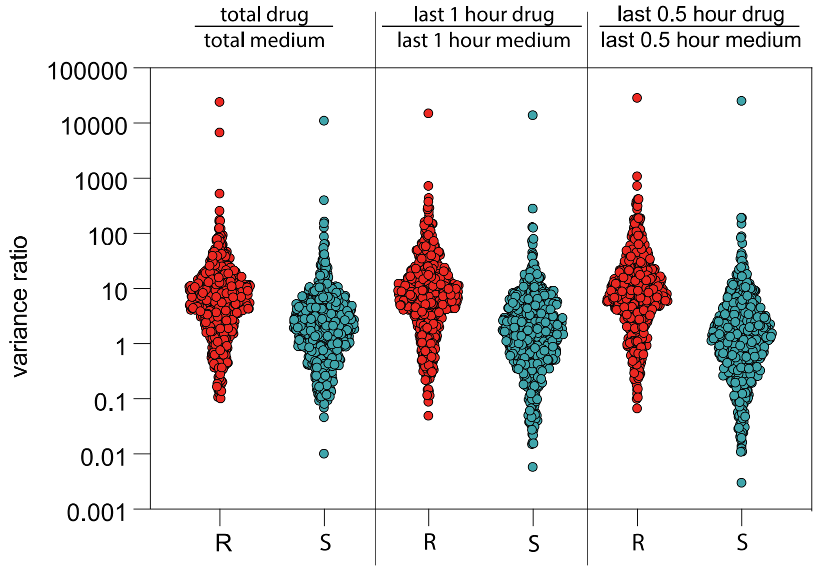

Supplementary Fig. 3 | Ratio analysis of the median variance of the entire 2-hour drug phase divided by the entire 2-hour medium phase or of parts of them (last 1h or last 0.5h of medium or drug phase, respectively). We show the ratios for 1485 recordings across 160 *E. coli* and *K. pneumoniae* isolates exposed to 32 µg/ml CRO. Visibly, neither ratio analysis allows a good separation of susceptible (S) and resistant (R) phenotypes demanding more sophisticated analysis tools and signal processing. Data are available in the source data file. The same recordings were used for sample performance calculations using ML algorithms depicted in Figure 4a.

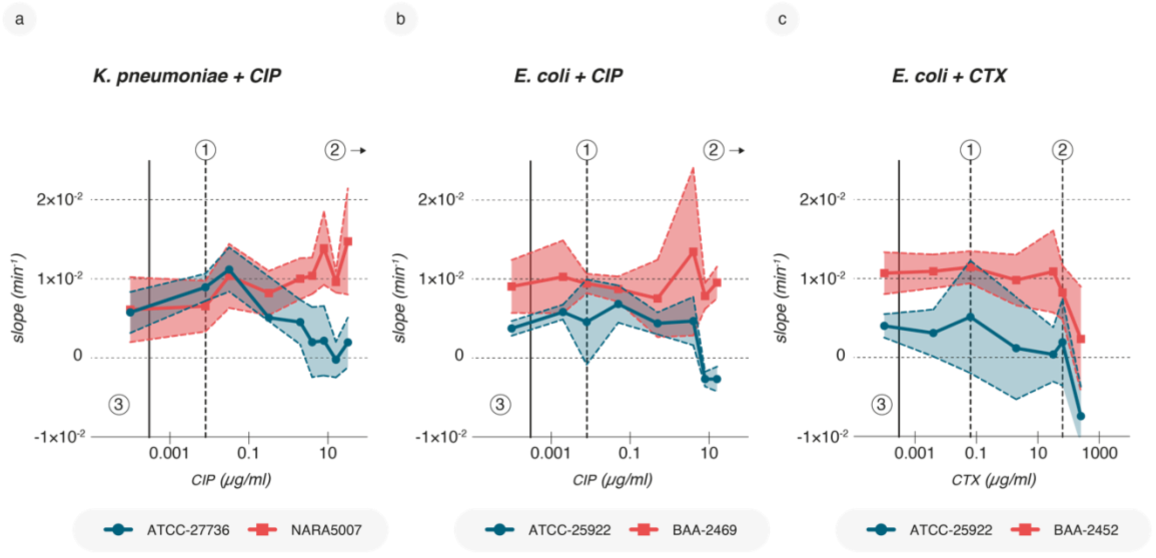

Supplementary Fig. 4 | Dose-responses a, CIP concentration-dependent slopes during the drug phase calculated by the formula $\log(x) = \log(C) + at$, where t is time, a is the slope of the common logarithm of the variance trend, and $\log(C)$ is the intercept. (1) and (2) represent the MICs of ATCC-27736 and NARA5007, respectively, while (3) represents the control without CIP, **b** similar to **a** for *E. coli* and different CIP concentrations. Shown are ATCC-25922 and BAA-2469 with their MICs at (1) and (2) and control without CIP (3), higher concentrations for CIP were not feasible because of the limited solubility of CIP in 50% LB, **c,** CTX-dependent variance slopes for *E. coli* ATCC-25922 and BAA-2452 with respective MICs (1,2) and control without CTX (3). For each concentration, at least 3 experiments were performed. Shown are the mean and SD, n =3. Data are available in the source data file.

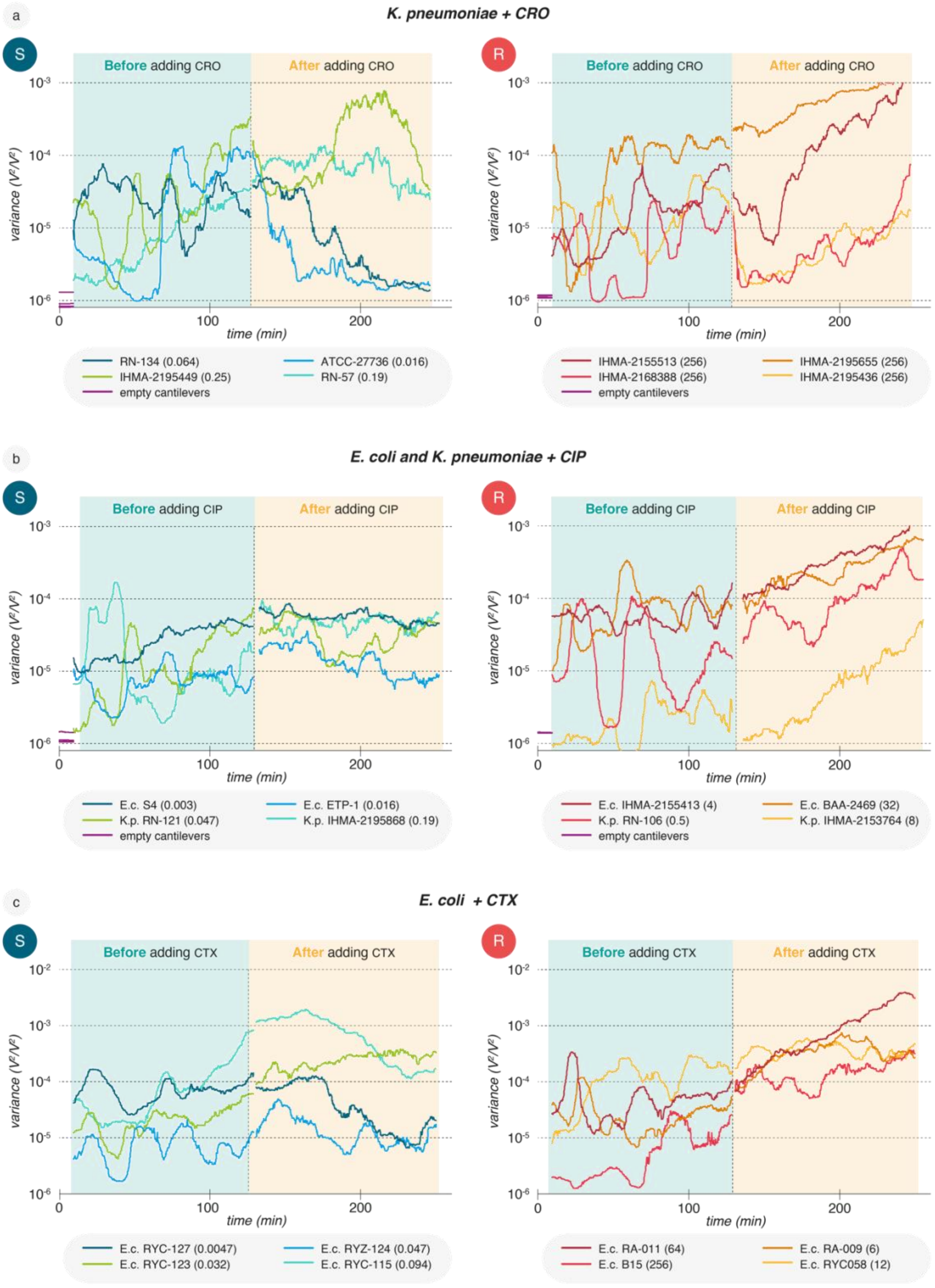

Supplementary Fig. 5 | Strain variability in nanomotion AST recordings showing the variance over time for different clinical *E. coli* and *K. pneumoniae* isolates. In each experiment, strains were attached to a cantilever and recorded in 50% LB for 2h (green shaded background) and then exposed to **a,** 32g/ml ceftriaxone (CRO), **b,** 8 µg/ml ciprofloxacin (CIP) or **c,** 32 µg/ml cefotaxime (CTX) for another 2 hours (orange shaded background).

Each strain's MIC (µg/ml) is indicated in brackets. Shown are representative recordings for each strain. Data are available in the source data file.

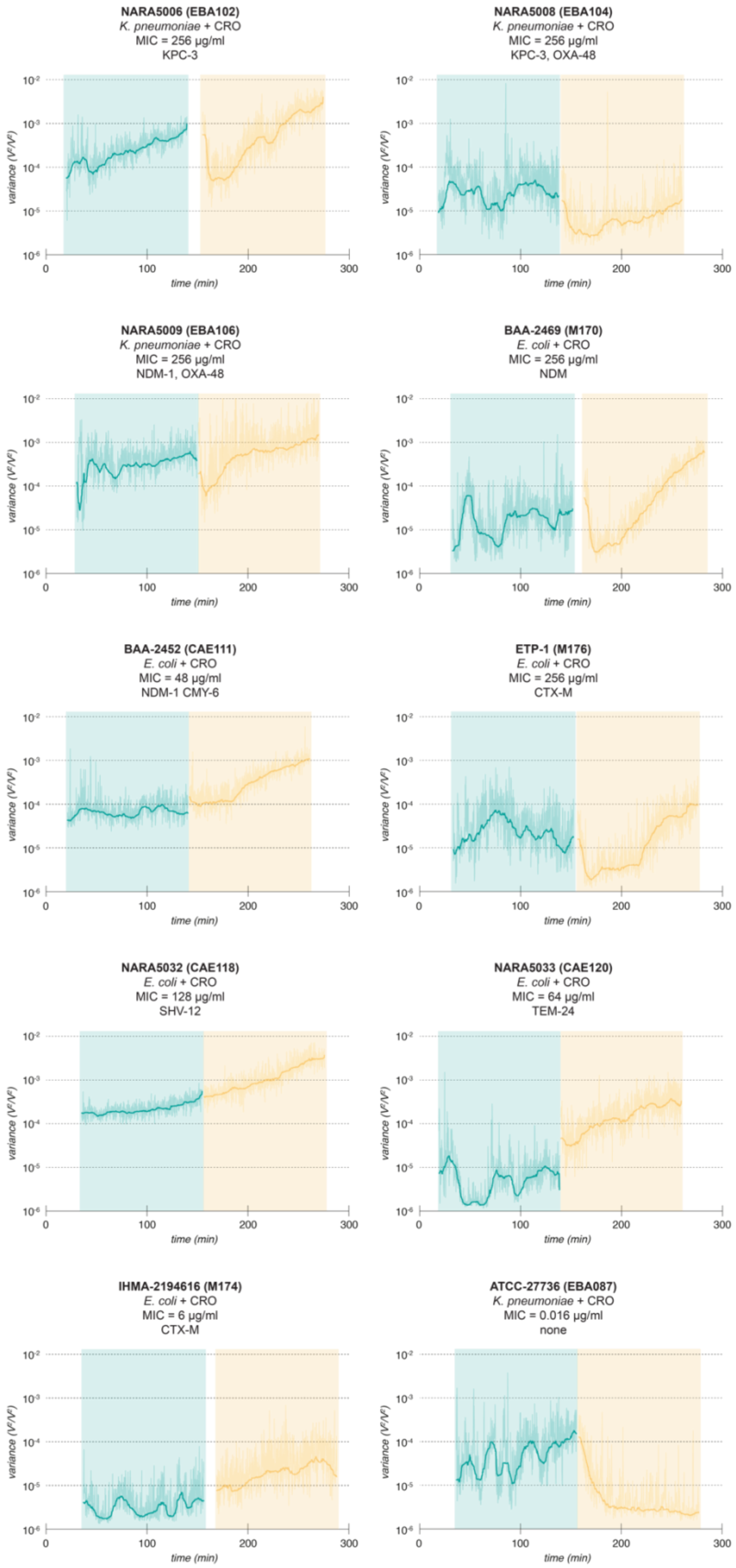
NARA5006 (EBA102)
K. pneumoniae + CRO
MIC = 256 µg/ml
KPC-3
NARA5008 (EBA104)
K. pneumoniae + CRO
MIC = 256 µg/ml
KPC-3, OXA-48
NARA5009 (EBA106)
K. pneumoniae + CRO
MIC = 256 µg/ml
NDM-1, OXA-48
BAA-2469 (M170)
E. coli + CRO
MIC = 256 µg/ml
NDM
BAA-2452 (CAE111)
E. coli + CRO
MIC = 48 µg/ml
NDM-1 CMY-6
ETP-1 (M176)
E. coli + CRO
MIC = 256 µg/ml
CTX-M
NARA5032 (CAE118)
E. coli + CRO
MIC = 128 µg/ml
SHV-12
NARA5033 (CAE120)
E. coli + CRO
MIC = 64 µg/ml
TEM-24
IHMA-2194616 (M174)
E. coli + CRO
MIC = 6 µg/ml
CTX-M
ATCC-27736 (EBA087)
K. pneumoniae + CRO
MIC = 0.016 µg/ml
none
variance (V²/V²)
time (min)
0
100
200
300

Supplementary Fig. 6 | Strain variability in nanomotion AST recordings showing the variance over time for different *E. coli* and *K. pneumoniae* clinical isolates. In each experiment, strains were attached to a cantilever and recorded in 50% LB for 2h (green) and then exposed to 32g/ml CRO for another 2 hours (orange). The variance plots of one sample is shown (3 technical replicates, in brackets in the title for each graph were combined), and the mean of the trendline and the mean variances every ten seconds are shown. We also indicate the type of beta-lactamase of each strain. Various nanomotion responses could be affected by the resistance mechanisms conferred by the different types of beta-lactamase. However, other factors are likely. MICs were determined by MIC strip. Data are available in the source data file.

a

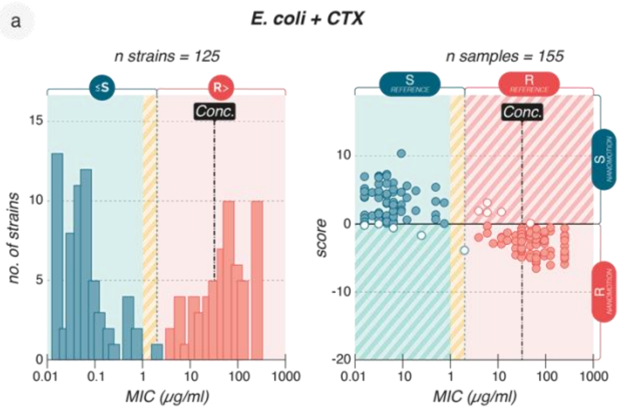

b

Metric	CTX
no. of recordings	*155*
True positive	*66*
True negative	*78*
False positive	*5*
False negative	*6*
accuracy (%)	***92.9***
sensitivity (%)	***91.7***
specificity (%)	***94.0***
no. of SPs	***5***

Supplementary Fig. 7 | Developing a classification model for *E. coli* + CTX. a, Left: To develop a model for CTX on 4-hour nanomotion recordings, the MIC distribution of 125 *E. coli* isolates was analysed at 32 µg/ml CTX (conc. dashed line). Right: Classification according to nanomotion and a pareto-optimal 5-SP model trained on 155 samples, with MIC strip serving as a reference. **b**, performance reporting accuracy [(TP+TN)/n], sensitivity [TP/(TP+FN)], and specificity [TN/((TN+FP)]. Data are available in the source data file. Single SP values and scores are available in Supplementary Data file 2.

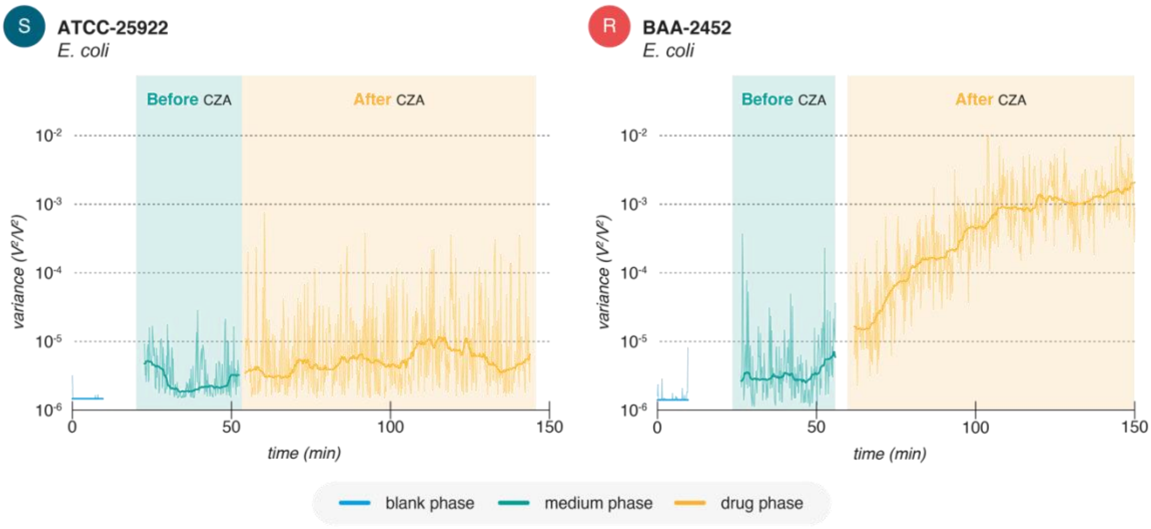

Supplementary Fig. 8 | Nanomotion recording at 37ºC as input for 2-hour based classification models for CZA. 2-hour nanomotion recordings with a 0.5-hour medium phase (50% LB medium) followed by a 1.5-hour drug phase with 32 µg/ml CAZ and 4 g/ml AVI (+ CZA) for the *E. coli* reference strains ATCC-25922 (S, susceptible) and BAA-2452 (R, resistant). Data are available in the source data file.

Supplementary Text

Development of susceptibility and resistance classification models using advanced signal processing of nanomotions and machine learning techniques

Supplementary Methodology

Premise and raw nanomotion signal

The classification algorithm in the nanomotion AST is based on a machine learning approach, employing a parametrized function that maps features of the classified object (vibrations of a microcantilever with attached bacteria) to the appropriate class (susceptible or resistant). The measurement process involves recording signals in two phases: the medium phase captures 2 hours of signals in the medium, while the drug phase records 2 hours of signals in the presence of a single specific drug concentration. This 4-hour signal is utilized to classify bacteria as susceptible or resistant. In one model for CZA the recordings are reduced to 2 hours comprising 0.5 hours of medium phase and 1.5 hours of drug phase.

Recording at 60,000 samples per second results in 864 million samples over 4 hours (2 hours: 432 million samples). Using the raw signal for classification presents challenges due to its size and the presence of irrelevant information. Factors like humidity, temperature, acoustic noise, and mechanical vibrations further complicate the signal. To address these issues, a feature extraction algorithm is necessary. This algorithm extracts real-number features, termed signal parameters (SP), which are utilized by the classification algorithm to accurately classify attached cells.

Definition of concrete mathematical values (features or signal parameters (SP)) from transformed signals

Feature extraction is a crucial step in machine learning, as the classification model relies on these extracted features to establish meaningful relationships between input features and classification outcomes. To extract relevant features (SPs), prior knowledge about the classification objects is essential.

Previous studies have explored relationships between signal variance and cell vibrations[1-3]. Some research focused on estimating the power spectral density of cantilever vibrations and linking flicker noise changes to cell vibrations[4,5]. These findings, however, were often obtained in highly controlled laboratory environments. Diagnostic laboratories in hospitals, in contrast, are much noisier, making classifying cantilever vibrations signals considerably more challenging. In this real-world context, fitting theoretical noise models necessitates robust optimization techniques and SPs based on robust statistics.

Our solution utilizes SPs derived from power spectral density, quantile spectrum, and multi-fractal detrended analysis[6]. The first two methods focus on frequency domain signal properties, while the third method employs a multiscale approach, estimating statistics in the time domain.

Frequency domain SPs are derived from time series of signal estimators. These estimators, derived from power spectrum or quantile spectrum, represent various aspects of the raw signal and are considered new signals themselves.

PSD derived SPs (Spectral SPs)

Estimators based on power spectral density are calculated from characteristic points and the shape of the spectrum (Supplementary Fig. 9). The power spectral density, calculated using the Welch method[7] of periodogram averaging, involves computing periodograms from consecutive intervals of the signal. Overlapping intervals, with an optimal overlap of half the interval, are allowed. Before computing the periodogram, the signal is detrended within each interval using linear detrending. This detrending involves fitting a linear equation to the signal within the interval, subtracting this linear trend from the signal. In the presented solution, power spectrum estimation occurs every 2 minutes. The low-frequency part of the spectrum, dominated by flicker noise, represents the cell vibrations signal most prominently.

For each spectrum, two parameters of the flicker noise model are calculated. They are estimated by fitting

$$PSD(f) = \frac{N_0}{f^\alpha}$$

noise model to the part of the spectrum ranging from 20 – 200 Hz. The results of fitting are N_0, alpha signal estimators.

Nevertheless, the low-frequency segment of the spectrum is susceptible to both low-frequency noise and drift due to environmental factors. These elements can adversely affect fitting accuracy. To mitigate this, estimators based solely on pure geometric spectrum properties are computed. These geometric properties are specific points indicating parts of the spectrum where the influence of cell vibrations, cantilever characteristics, and background noise is evident. This approach relaxes the rigid constraints of specific noise models, rendering the resulting signal estimators more robust. The identified points are as follows:

- **Local Minimum Point (fmin, pmin):** This point reflects the balance between flicker, white, and thermomechanical noise. The position and height of the minimum indicate where thermomechanical noise begins to dominate the power spectrum, serving as a reference.
- **Local Maximum Point (fmax, pmax):** This point is closely related to thermomechanical noise, influenced by cantilever mechanical properties and its interaction with the medium.
- **Midpoints (fleft, fright, pleft, pright):** These points, positioned midway between the local minimum and maximum, offer a detailed insight into thermomechanical noise. They measure the asymmetry of the thermomechanical noise peak, potentially caused by additional cantilever excitation due to vibrating cells.

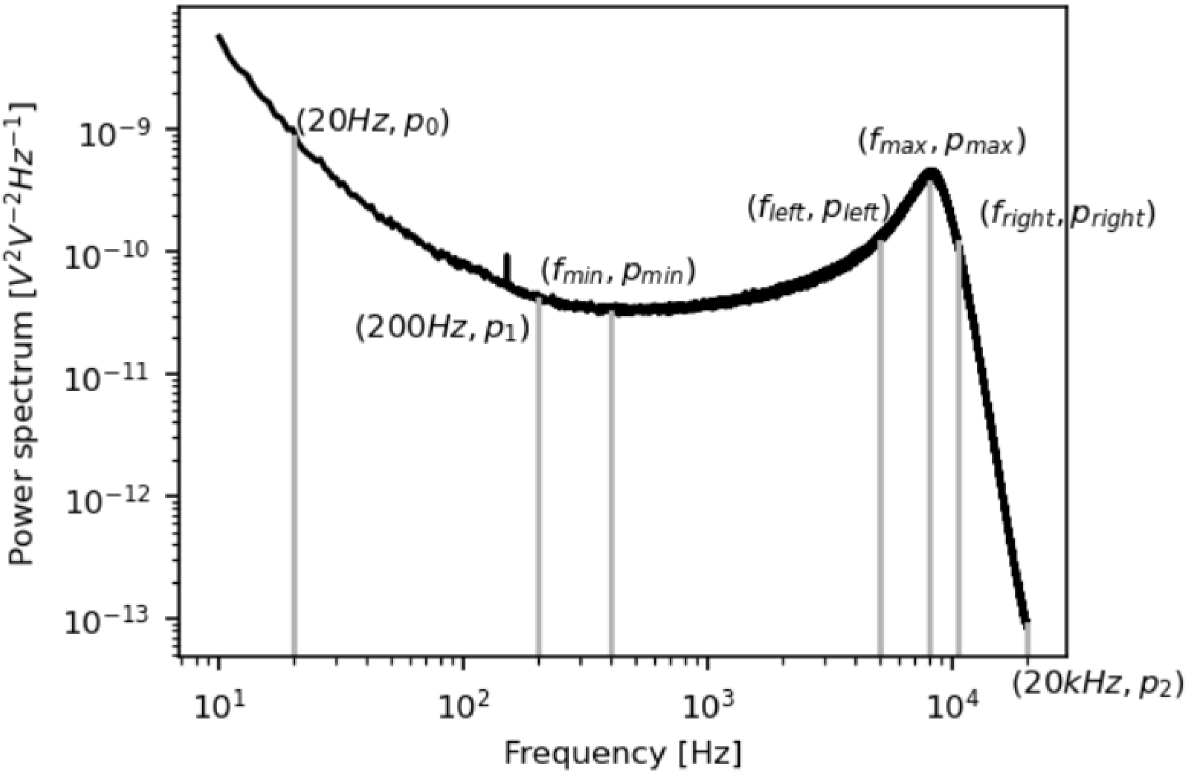

Supplementary Fig. 9 | Characteristic points in the power spectral density (PSD) of the cantilever vibration signal used to define SPs

The extracted points are utilized to compute noise levels within specific frequency ranges defined by these points. Five ranges are established: (20 Hz, *fmin*), (*fmin*, *fleft*), (*fleft*, *fmax*), (*fmax*, *fright*), and (*fright*, 20 kHz), denoted as *N1* to *N5*. Additionally, noise levels are measured in static frequency ranges. For this reason the area under the curve is calculated for 20 equidistant log-scale frequency ranges (20-28, 28-39, … , 224-316, 316-447, … , 10,023-14,158, 14,158-20,000 Hz), referred to as *NE1* to *NE20*. This method provides detailed information about noise distribution accommodating multiple low frequency ranges and electronic noise impact of the device itself.
All noise levels are also available in normalized form related to the noise level from 20 Hz to *fright* frequency (*normNi* and *normNEi* for every *i* *Ni*/(*N1*+*N2*+*N3*+*N4*+*N5*) and *NEi/*(*N1*+*N2*+*N3*+*N4*+*N5*)). In this way 60 time series of signal spectral estimators are calculated.

Quantile SPs

The Welch method of PSD estimation can be extended by calculating periodogram percentiles[8]. This statistical approach's robustness, compared to the Welch method, is emphasized in this study for some of the classification models. In this application, multiple periodogram percentiles are computed, introducing an additional dimension for analyzing cell vibration signals. The following Supplementary Fig. 10 displays the percentile spectra for the 10th, 50th, and 90th percentiles, alongside the standard power spectral density.

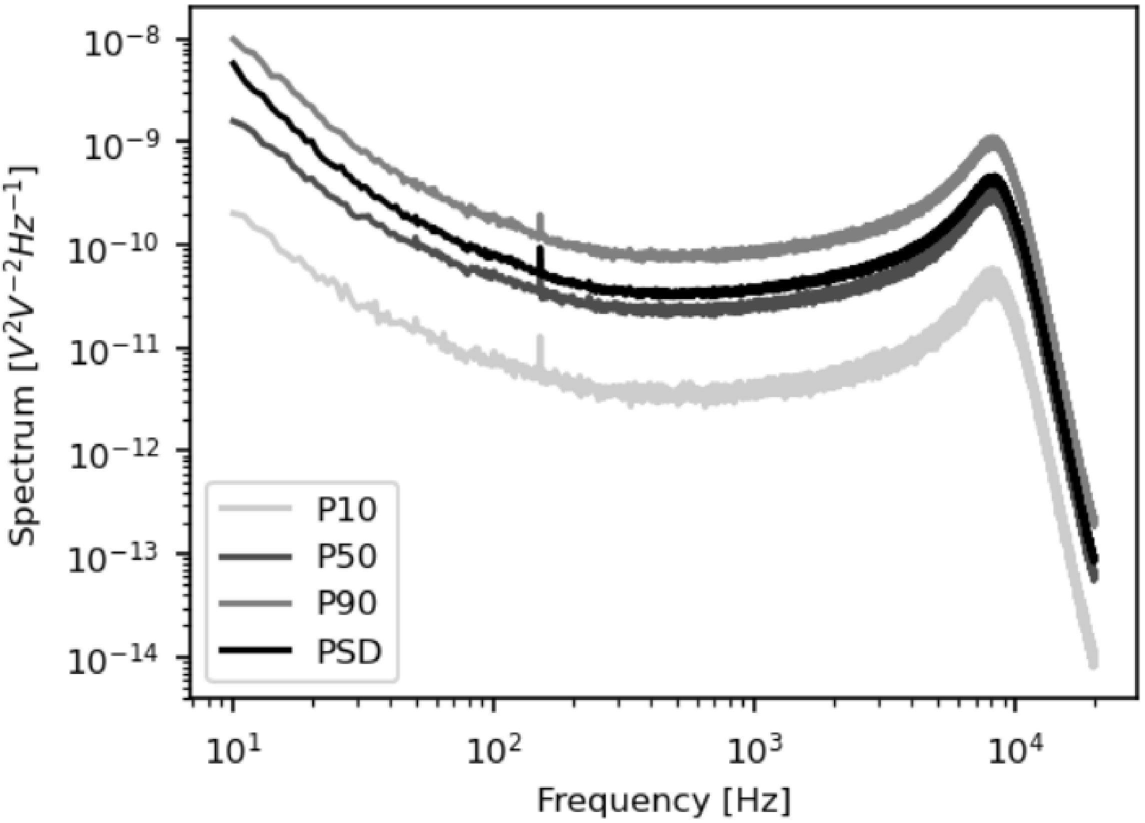

Supplementary Fig. 10 | Comparison of quantile spectrum and power spectrum for three percentiles (10th, 50th, 90th)

Percentiles are computed not only for periodogram bins but also for specific frequency ranges, aiming to quantify rarely correlated events that are filtered out by periodogram averaging. Further in the text this statistic will be called quantile spectrum. The frequency ranges used for the quantile spectrum are

0-10, 10-100, 100-200, 200-400, 400-1000, 1000-2000, 2000-4000, 4000-5000, 5000-6000, 6000-7000, 7000-8000, 8000-10000 Hz. Within these ranges, 10 percentiles are estimated (10th, 20th, … , 90th).
These percentiles (such as the 50th percentile - p_{50}) serve as signal estimators and are further used to construct derived signal estimators like spikiness e.g. (p_{60}-p_{40})/(p_{90}-p_{10}) or the symmetry measure, e.g., (p_{50}-p_{10})/(p_{90}-p_{50}). The symmetry measure identifies correlated spurious events buried in a correlated signal.
In Supplementary Fig. 11, a simulated signal of this nature is depicted. Neither the histogram (Supplementary Fig. 12) nor the power spectral density (PSD) (Supplementary Fig. 13) can distinguish the simulated signals. However, the difference becomes evident in the symmetry spectrum (Supplementary Fig. 14), calculated from the quantile spectrum. This simulation illustrates the kind of information that can be observed using the quantile spectrum.

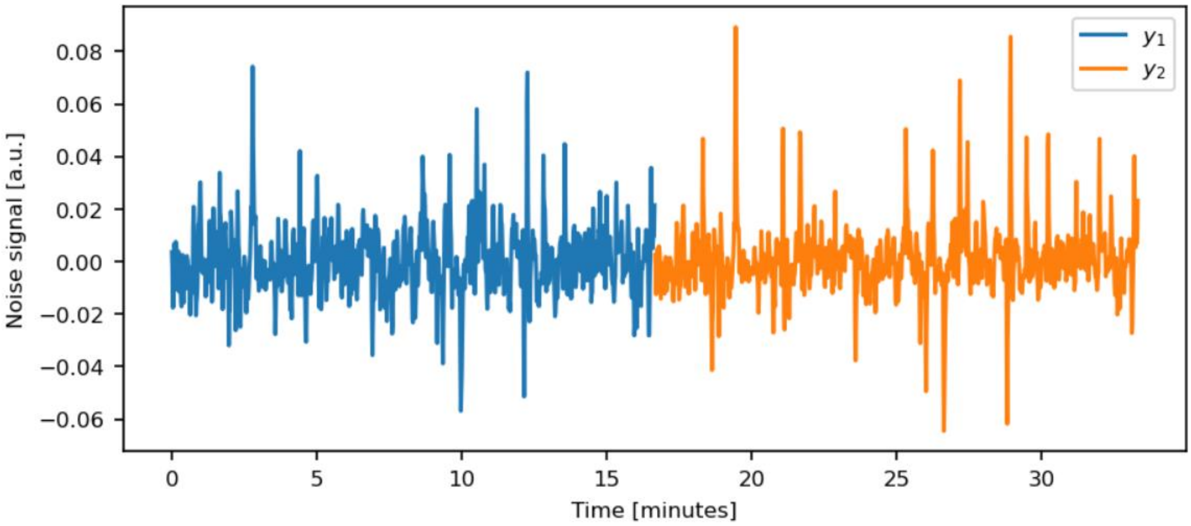

Supplementary Fig. 11 | Simulated random signals being weighted sum of Gaussian noise and random spurious events

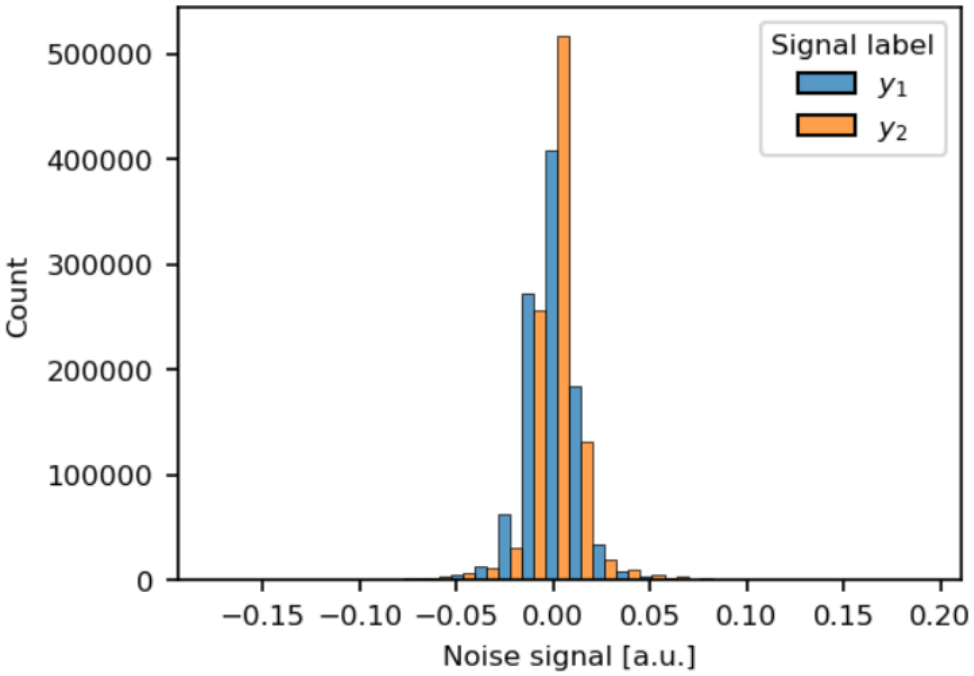

Supplementary Fig. 12 | Histograms of the simulated random signals

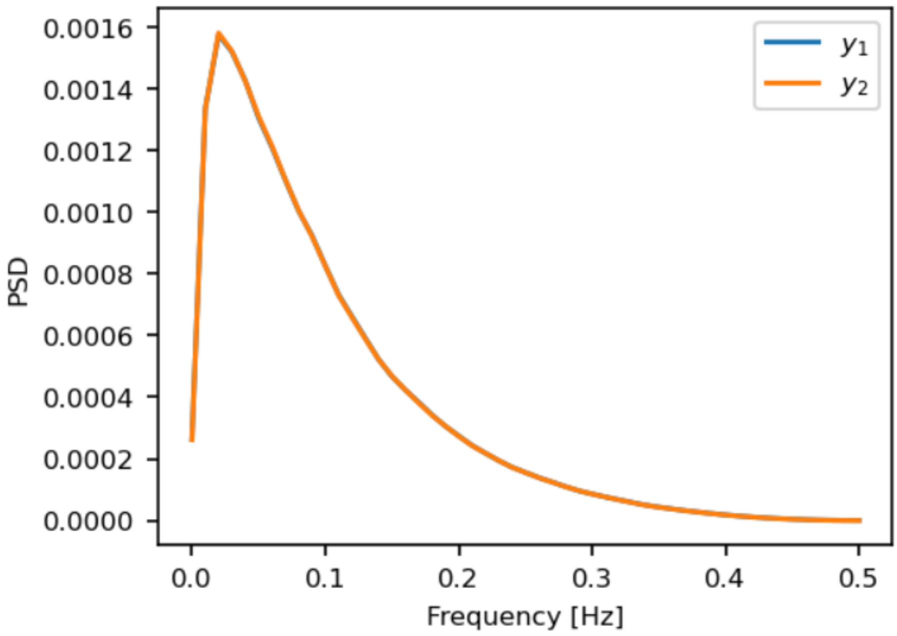

Supplementary Fig. 13 | PSD of the simulated random signals

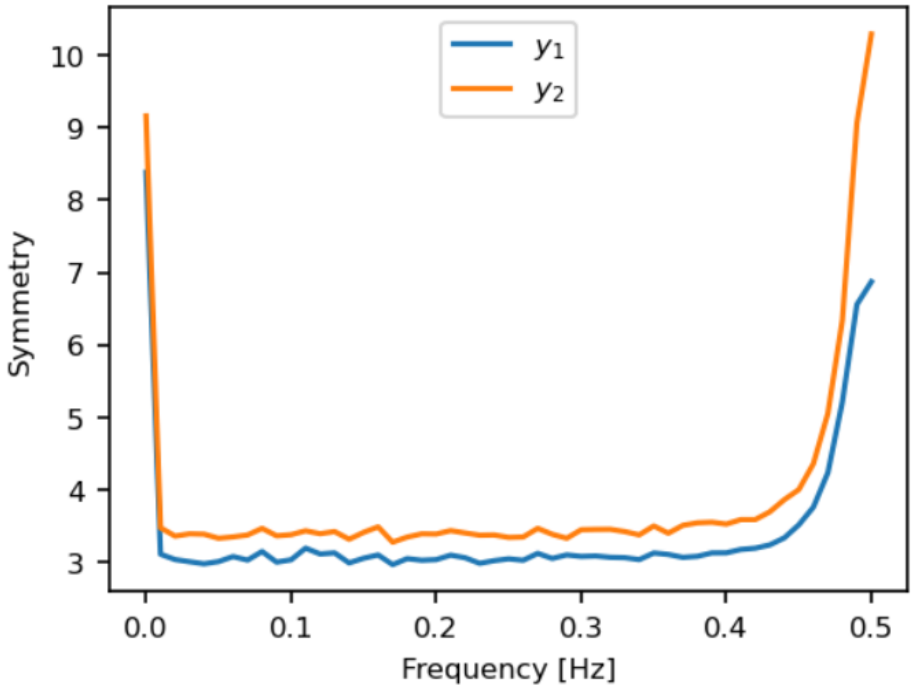

Supplementary Fig. 14 | Symmetry of quantile spectrum for simulated random signals

In the context of Welsh periodograms, the expected probability distribution follows a Chi-squared distribution. However, spurious events in the signal alter this distribution. Consequently, introducing a measure of divergence between the Chi-squared and empirical distribution, estimated from quantiles, becomes pertinent. In this application, the Jensen-Shannon divergence[9] and its square root, the Jensen-Shannon distance, are employed to quantify the divergence from the Chi-squared distribution. Additionally, a 3rd order polynomial fit to quantiles is utilized to introduce shape indicators in the form of fitted parameter values. These shape indicators, along with Jensen-Shannon divergence and distance, serve as additional signal estimators. This approach results in 115 signal estimators computed every 5 minutes.

The frequency domain time series of signal estimators undergo a transformation into SPs. This transformation involves splitting the data into intervals lasting from 10 to 60 minutes. Within each interval, diverse statistics such as moments, percentiles, and generalized means are computed. Additionally, time-dependent functions like polynomials and exponentials are fitted. These statistics become SPs, along with the parameters from the fitted time-dependent functions. Furthermore, these SPs are combined to create additional SPs through the calculation of various ratios and differences. This approach establishes relationships between SPs measuring cell vibrations and those gauging the background noise.

MF-DFA SPs

The approach to time-domain-related SPs draws inspiration from the multifractal detrended fluctuation analysis method[6]. This method, an extension of the earlier detrended fluctuation analysis[10], assesses the signal's self-affinity across various scales defined by exponents of generalized means. In this presented approach, instead of calculating the generalized Hurst exponent[6], the generalized means are directly utilized as SPs. The process of calculating MF-DFA SPs involves using the first and last 20 minutes of the signal for analysis. Within these two time periods, the signal is integrated and segmented into detrending periods of specific lengths (ranging from 1.17 to 300 seconds). This segmentation results in 1024 periods of 1.17 seconds length, 512 periods of 2.34 seconds length, and so on, up to 4 periods of 300 seconds length. For each period length, a 2nd order polynomial is fitted and employed to detrend the integral of the signal. Subsequently, generalized means of the detrended integrals are computed to derive SPs. The definition of the generalized mean with real exponent *q* is as follows:

for q=0

$$F_0 = exp\left(\frac{1}{N}\sum_{i=1}^{N} ln(|x_i|)\right)$$

for $q \neq 0$

$$F_q = \left(\frac{1}{N}\sum_{i=1}^{N} |x_i|^q\right)^{\frac{1}{q}}$$

where x_i is a ith sample of signal counting N samples.

The generalized means serve to discern subtle variations in the probability density function (PDF) shape of the random signal. To illustrate, white noise with a PDF comprising a blend of two Gauss functions with distinct standard deviations was simulated. After 60 minutes, the standard deviation of the first Gaussian was doubled, while the second remained unchanged (see Supplementary Fig. 15). This signal alteration is undetectable through traditional means such as histograms (Supplementary Text Fig.8) or standard deviation (calculated with a generalized mean exponent of 2 – refer to Supplementary Fig. 17). However, it is discerned by the geometric mean (with an exponent of 0 – see Supplementary Fig. 17). Changes in the PDF may manifest differently across various time scales, and these shifts are captured by MF-DFA SPs. These SPs explore similar aspects of the signal as quantile SPs but function in the time domain.

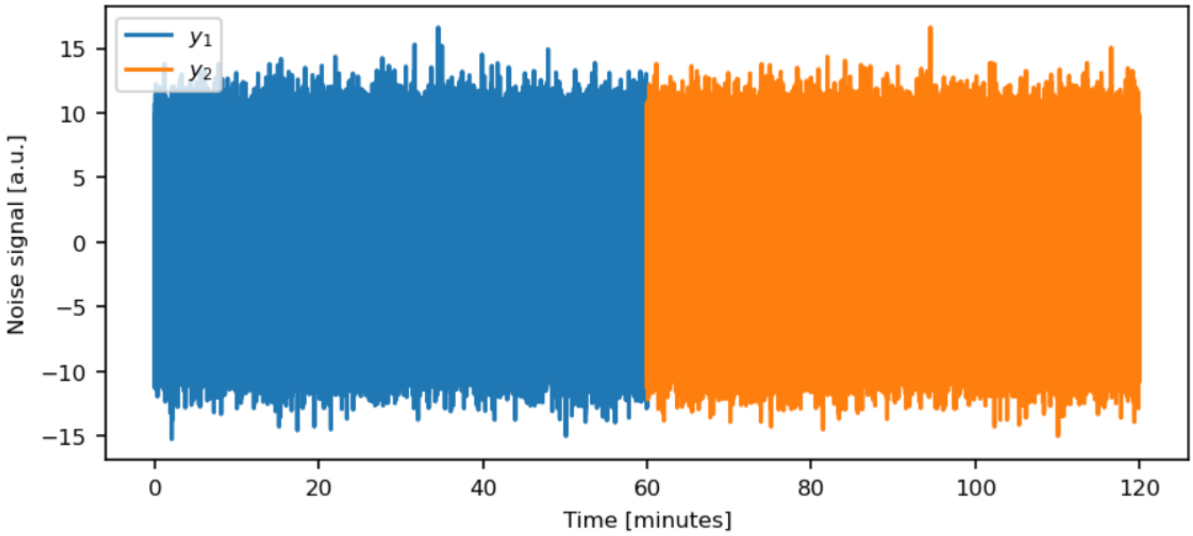

Supplementary Fig. 15 | Random signal with PDF shape changed.

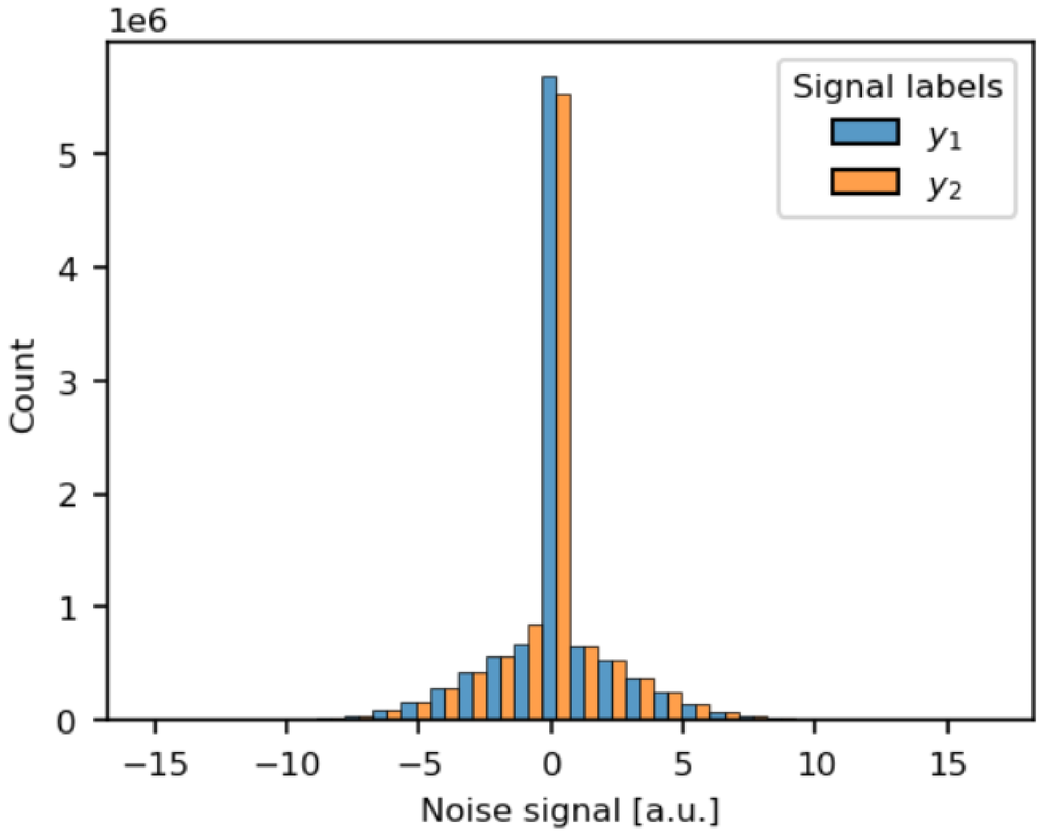

Supplementary Fig. 16 | Histograms of the signal from Supplementary Fig. 15.

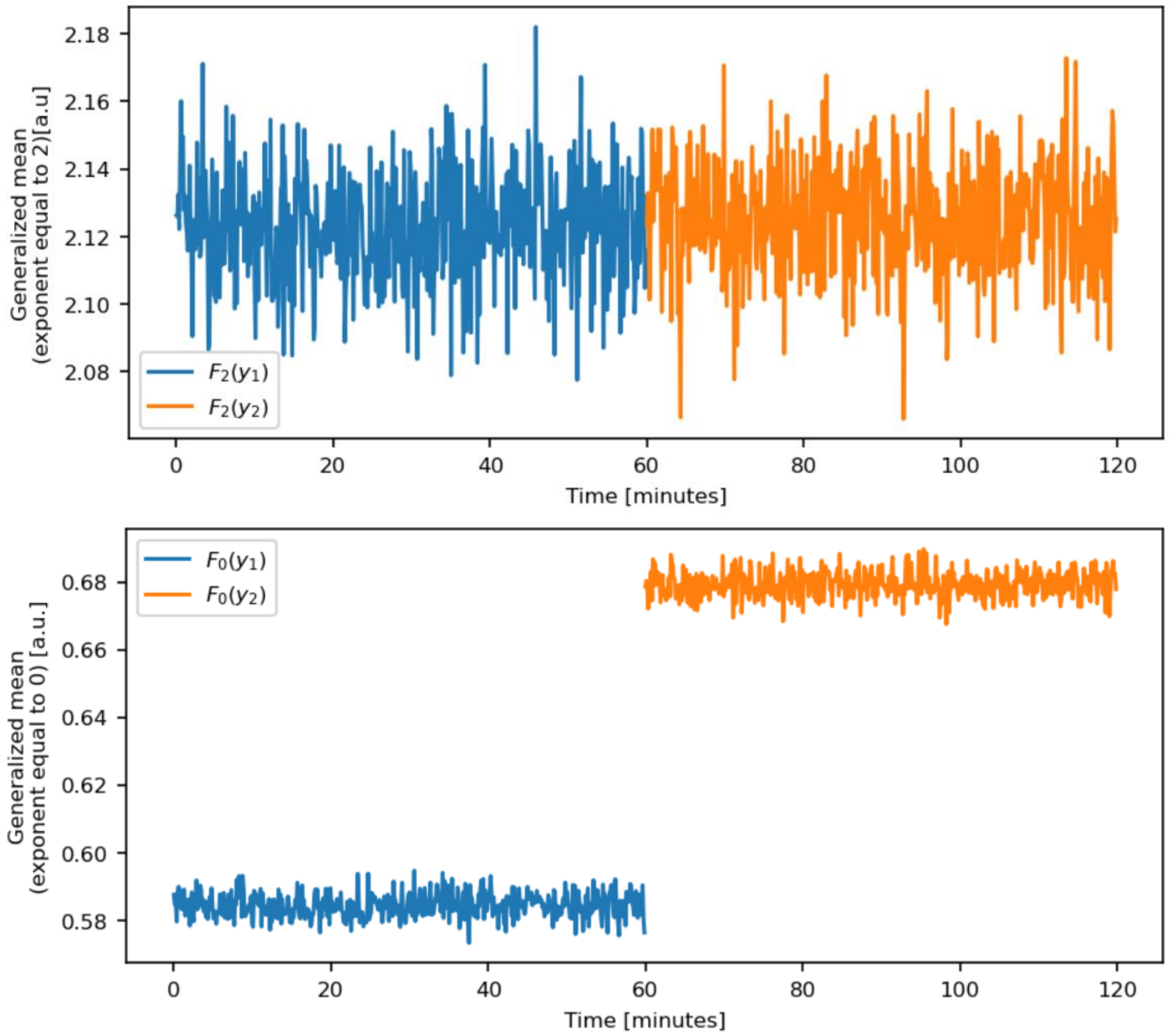

Supplementary Fig. 17 | Two generalized means F_2 (standard deviation) and F_0 (geometric mean). Geometric mean detects changes in the signal PSD while basic standard deviation not.

In this application, means are calculated for 21 exponents (-10, -8, -6, -4, -2, -1, -0.8, -0.6, -0.4, -0.2, 0, 0.2, 0.4, 0.6, 0.8, 1, 2, 4, 6, 8, 10) and 9 detrending periods, resulting in 189 SPs associated with various exponent-detrending period pairs. The first and last 20 minutes of the drug phase are utilized for MF-DFA SP calculations. Only ratios of corresponding means (with the same exponent q and detrending period length) are computed. These ratios are then combined into all possible combinations to relate various exponent-detrending period pairs. For each AST experiment, hundreds of thousands of SPs are calculated, and a feature selection algorithm is employed to identify the optimal parameters. This selection method involves a classification algorithm, an optimality criterion, and a cross-validation technique.

Classification Model based on SPs

The logistic regression algorithm[11] is employed for classification. SPs are weighted and combined with an offset to compute a score value. This score serves as input for a logistic function, mapping real numbers to values between 0 and 1. The weights of SPs and the offset are determined by maximum likelihood estimation through model training using labeled data (label 0 means resistant label 1 means susceptible). Logistic regression's primary advantage lies in its minimal parameter count, equal to the number of SPs plus 1 (for the offset). Strains are labeled as susceptible or resistant based on the reference AST method.

The feature extraction phase generates thousands of SPs potentially containing information about cell vibrations and background noise. However, the optimal representation of this data is determined by a feature selection algorithm. This algorithm requires defining the optimal set of SPs, considering two criteria: classification accuracy and the number of SPs. The approach involves multicriteria optimization, where accuracy is maximized while minimizing the number of SPs. The concept of Pareto optimality[12] is applied: models with maximal accuracy and the fewest SPs are retained. If adding or removing a SP improves or maintains accuracy, the model with the fewer parameters is selected. This process ensures the selection of models with a minimal number of SPs. Model accuracy is evaluated through cross-validation.

Model selection based on Cross-validation and final set of SPs for each model

Cross-validation is a method used to assess the performance of a classification algorithm. In this application, model accuracy is the key performance metric. The 3-fold cross-validation procedure was repeated 300 times. The dataset was divided randomly into three equal sets. The classification algorithm was trained on two sets, and its performance, measured by accuracy, was calculated on the third set. By repeating this process 300 times and averaging the results, 900 estimates of model performance were obtained, allowing for a reliable determination of model accuracy.

The feature selection process involves evaluating SPs one by one through cross-validation to determine model accuracy. Each parameter is added to the model, and the one resulting in the highest accuracy is chosen. This process continues, adding subsequent parameters, until no further improvement in accuracy is observed. This approach aligns with the Pareto optimality concept, where adding more parameters may worsen both the number of parameters and accuracy. Once forward selection is complete, backward selection starts, removing parameters one by one until only one optimal model, meeting the Pareto optimal criteria, remains. The presented results are obtained for logistic regression classification algorithm for which the number of model parameters fitted in the training process is equal to the number of SPs plus offset. Since the feature selection leads to models with just few SPs, the model complexity is low while the model is still able to classify strains with high accuracy.

The SPs of the models presented in this study are organized in the following tables 1 to 6 after their appearance in the main text. The SPs are ranked by their importance in the specific model. This means that subsequent Pareto optimal models are created by eliminating the SP with the highest index (for example, SP4 in the model for CRO).

Supplementary Results: SPs for the classification models presented in the study

Following the steps presented in the Supplementary Methodology, the SPs of the models presented in this study are organized in the following tables 1 to 6 after their appearance in the main text. The SPs are ranked by their importance in the specific model. This means that subsequent Pareto optimal models are created by eliminating the SP with the highest index (for example, SP4 in the model for CRO).

Supplementary Table 1 | MF-DFA based SPs for the model *K. pneumoniae* + CIP

Parameter	Period-exponent pair	Phase	Time range [min]	Generalized mean exponent	Detrending period [s]	Pairs relation
SP1	PE1	Drug	100-120	0.8	5	PE1/PE2
	PE2	Drug	0-20	0.8	5	
SP2	PE1	Drug	100-120	-0.8	150	PE1/PE2
	PE2	Drug	0-20	-0.8	150	
SP3	PE1	Drug	100-120	2	5	(PE1 PE4)/(PE2 PE3)
	PE2	Drug	0-20	2	5	
	PE3	Drug	100-120	0.8	9	
	PE4	Drug	0-20	0.8	9	
SP4	PE1	Drug	100-120	-10	150	(PE1 PE4)/(PE2 PE3)
	PE2	Drug	0-20	-10	150	
	PE3	Drug	100-120	-10	1	
	PE4	Drug	0-20	-10	1	

Supplementary Table 2 | PSD based SPs for the model *E. coli/K. pneumoniae* + CRO

Parameter	Interval	Phase	Time range for PSD calculation measured from the beginning of the phase	PSD signal estimator	Frequency range [Hz]	Normalized	Interval statistic	Intervals relation
SP1	T1	Drug	90 -120	Integral	20-28	no	median	T1/T2
	T2	Drug	0-30	Integral	20-28	no	median	
SP2	T1	Drug	90-120	Integral	14159-20000	yes	median	T1/T2
	T2	Drug	60-90	Integral	14159-20000	yes	median	
SP3	T1	Medium	90-120	Integral	1783-2518	yes	median	T1/T2
	T2	Medium	0-120	Integral	1783-2518	yes	median	
SP4	T1	Drug	90-120	Integral	28-40	yes	median	T1/T2
	T2	Drug	0-120	Integral	28-40	yes	median	

Supplementary Table 3 | Quantile and MF-DFA based SPs for the model *E. coli* + CIP

Parameter	Time-frequency subspace	Phase	Time range for PSD calculation measured from the beginning of the phase	Quantile spectrum signal estimator	Frequency range [Hz]	Interval statistic	Subspaces relation
SP1	TF1	Drug	90 -120	Symmetry	0-10	mean	(TF1 TF4)/(TF2 TF3)
	TF2	Drug	90-120	Symmetry	5000-6000	mean	
	TF3	Drug	0-30	Symmetry	0-10	mean	
	TF4	Drug	0-30	Symmetry	5000-6000	mean	
SP2	TF1	Drug	90-120	20th percentile	200-400	mean	(TF1 TF4)/(TF2 TF3)
	TF2	Drug	90-120	20th percentile	10-100	mean	
	TF3	Drug	30-60	20th percentile	200-400	mean	
	TF4	Drug	30-60	20th percentile	10-100	mean	
SP3	TF1	Medium	60-90	30th percentile	10-100	mean	TF1/TF2
	TF2	Medium	60-90	30th percentile	0-10	mean	

Parameter	Period-exponent pair	Phase	Time range [min]	Generalized mean exponent	Detrending period [s]	Pairs relation
SP4	PE1	Drug	100-120	-0.4	9	PE1/PE2
	PE2	Drug	0-20	-0.4	9	

Supplementary Table 4 | Quantile and MF-DFA based SPs for the model *E. coli* + CTX

Parameter	Time-frequency subspace	Phase	Time range for PSD calculation measured from the beginning of the phase	Qantile spectrum signal estimator	Frequency range [Hz]	Interval statistic	Subspaces relation
SP1	TF1	Drug	90 -120	median	400-1000	mean	(TF1 TF4)/(TF2 TF3)
	TF2	Drug	90-120	median	100-200	mean	
	TF3	Drug	0-30	median	400-1000	mean	
	TF4	Drug	0-30	median	100-200	mean	
SP2	TF1	Drug	30-60	Symmetry	200-400	mean	(TF1 TF4)/(TF2 TF3)
	TF2	Drug	30-60	Symmetry	100-200	mean	
	TF3	Drug	0-30	Symmetry	200-400	mean	
	TF4	Drug	0-30	Symmetry	10-100	mean	
SP5	TF1	Drug	90-120	70th percentile	1000-2000	mean	(TF1 TF4)/(TF2 TF3)
	TF2	Drug	90-120	70th percentile	100-200	mean	
	TF3	Drug	0-30	70th percentile	1000-2000	mean	
	TF4	Drug	0-30	70th percentile	100-200	mean	

Parameter	Period-exponent pair	Phase	Time range [min]	Generalized mean exponent	Detrending period [s]	Pairs relation
SP3	PE1	Drug	100-120	10	75	(PE1 PE4)/(PE2 PE3)
	PE2	Drug	0-20	10	75	
	PE3	Drug	100-120	-0.6	2	
	PE4	Drug	0-20	-0.6	2	
SP4	PE1	Drug	100-120	-1	1	(PE1 PE4)/(PE2 PE3)
	PE2	Drug	0-20	-1	1	
	PE3	Drug	100-120	-2	5	
	PE4	Drug	0-20	-2	5	

Supplementary Table 5 | PSD based SPs for the 4-hours-model *E. coli* + CZA

Parameter	Interval	Phase	Time range for PSD calculation measured from the beginning of the phase	PSD signal estimator	Frequency range [Hz]	Normalized	Interval statistic	Intervals relation
SP1	T1	Drug	85-105	PSD local minimum	irrelevant	irrelevant	90th percentile	T1/T2
	T2	Drug	5-25	PSD local minimum	irrelevant	irrelevant	90th percentile	

Supplementary Table 6 | Quantile based SPs for the 2-hours-model *E. coli/K. pneumoniae* + CZA

Parameter	Time-frequency subspace	Phase	Time range for PSD calculation measured from the beginning of the phase	Quantile spectrum signal estimator	Frequency range [Hz]	Interval statistic	Subspaces relation
SP1	TF1	Drug	90 -120	Jensen-Shannon distance	0-10	25th percentile	TF1/TF2
	TF2	Drug	30-60	Jensen-Shannon distance	0-10	25th percentile	
SP2	TF1	Drug	90-120	The 1st coefficient of the 3rd order polynomial	10-100	Standard deviation	TF1/TF2
	TF2	Drug	60-90	The 1st coefficient of the 3rd order polynomial	100-200	Standard deviation	
SP3	TF1	Drug	90-120	Spikiness	1000-2000	Mean	TF1/TF2
	TF2	Drug	90-120	Spikiness	0-10	Mean	
SP4	TF1	Medium	15-30	The 3rd coefficient of the 3rd order polynomial	200-400	Standard deviation	TF1/TF2
	TF2	Medium	0-15	The 3rd coefficient of the 3rd order polynomial	100-200	Standard deviation	
SP5	TF1	Medium	15-30	Jensen-Shannon divergence	400-1000	Standard deviation	TF1/TF2
	TF2	Medium	0-15	Jensen-Shannon divergence	0-10	Standard deviation	
SP6	TF1	Medium	15-30	Jensen-Shannon distance	200-400	95th percentile	TF1

References

1 Longo, G. *et al.* Rapid detection of bacterial resistance to antibiotics using AFM cantilevers as nanomechanical sensors. *Nat Nanotechnol* **8**, 522-526 (2013). https://doi.org:10.1038/nnano.2013.120

2 Villalba, M. I. *et al.* Nanomotion Detection Method for Testing Antibiotic Resistance and Susceptibility of Slow-Growing Bacteria. *Small* **14** (2018). https://doi.org:10.1002/smll.201702671

3 Wu, S. *et al.* Quantification of cell viability and rapid screening anti-cancer drug utilizing nanomechanical fluctuation. *Biosensors and Bioelectronics* **77**, 164-173 (2016). https://doi.org:10.1016/j.bios.2015.09.024

4 Johnson, W. L., France, D. C., Rentz, N. S., Cordell, W. T. & Walls, F. L. Sensing bacterial vibrations and early response to antibiotics with phase noise of a resonant crystal. *Scientific Reports* **7** (2017). https://doi.org:10.1038/s41598-017-12063-6

5 Lissandrello, C. *et al.* Nanomechanical motion of Escherichia coli adhered to a surface. *Applied Physics Letters* **105** (2014). https://doi.org:10.1063/1.4895132

6 Kantelhardt, J. W. *et al.* Multifractal detrended fluctuation analysis of nonstationary time series. *Physica A: Statistical Mechanics and its Applications* **316**, 87-114 (2002). https://doi.org:10.1016/s0378-4371(02)01383-3

7 Welch, P. The use of fast Fourier transform for the estimation of power spectra: A method based on time averaging over short, modified periodograms. *IEEE Transactions on Audio and Electroacoustics* **15**, 70-73 (1967). https://doi.org:10.1109/tau.1967.1161901

8 Schwock, F. & Abadi, S. in *ICASSP 2021 - 2021 IEEE International Conference on Acoustics, Speech and Signal Processing (ICASSP)* 5165-5169 (2021).

9 Lin, J. Divergence measures based on the Shannon entropy. *IEEE Transactions on Information Theory* **37**, 145-151 (1991). https://doi.org:10.1109/18.61115

10 Peng, C. K. *et al.* Mosaic organization of DNA nucleotides. *Physical Review E* **49**, 1685-1689 (1994). https://doi.org:10.1103/PhysRevE.49.1685

11 Cramer, J. S. The Origins of Logistic Regression. *SSRN Electronic Journal* (2003). https://doi.org:10.2139/ssrn.360300

12 Pareto, V. *Cours d'Economie Politique*. Vol. Vol. II. Lausanne (1897).
