# Supplementary material for: Accurate and rapid antibiotic susceptibility testing using a machine learning-assisted nanomotion technology platform
Source: Nat Commun. 2024 Mar 18;15:2037. doi: 10.1038/s41467-024-46213-y (PMC10948838; doi:10.1038/s41467-024-46213-y)
Supplement: Supplementary file 3 — Description of Additional Supplementary Files [file 41467_2024_46213_MOESM3_ESM.pdf]

Description of Additional Supplementary Files

Supplementary Data Legends

Supplementary Data 1: Strains used for training and testing classification models

Supplementary Data 2: Experiments used for training and testing per model
