# Supplementary material for: Accurate and rapid antibiotic susceptibility testing using a machine learning-assisted nanomotion technology platform
Source: Nat Commun. 2024 Mar 18;15:2037. doi: 10.1038/s41467-024-46213-y (PMC10948838; doi:10.1038/s41467-024-46213-y)
Supplement: Supplementary file 6 — Reporting Summary [file 41467_2024_46213_MOESM6_ESM.pdf]

nature portfolio

Corresponding author(s): Alexander Sturm

Last updated by author(s): Jan 19, 2024

Reporting Summary

Nature Portfolio wishes to improve the reproducibility of the work that we publish. This form provides structure for consistency and transparency in reporting. For further information on Nature Portfolio policies, see our Editorial Policies and the Editorial Policy Checklist.

Statistics

For all statistical analyses, confirm that the following items are present in the figure legend, table legend, main text, or Methods section.

n/a	Confirmed	
☐	☒	The exact sample size (*n*) for each experimental group/condition, given as a discrete number and unit of measurement
☐	☒	A statement on whether measurements were taken from distinct samples or whether the same sample was measured repeatedly
☐	☒	The statistical test(s) used AND whether they are one- or two-sided<br>*Only common tests should be described solely by name; describe more complex techniques in the Methods section.*
☒	☐	A description of all covariates tested
☒	☐	A description of any assumptions or corrections, such as tests of normality and adjustment for multiple comparisons
☐	☒	A full description of the statistical parameters including central tendency (e.g. means) or other basic estimates (e.g. regression coefficient) AND variation (e.g. standard deviation) or associated estimates of uncertainty (e.g. confidence intervals)
☐	☒	For null hypothesis testing, the test statistic (e.g. F, t, r) with confidence intervals, effect sizes, degrees of freedom and P value noted<br>*Give P values as exact values whenever suitable.*
☒	☐	For Bayesian analysis, information on the choice of priors and Markov chain Monte Carlo settings
☒	☐	For hierarchical and complex designs, identification of the appropriate level for tests and full reporting of outcomes
☒	☐	Estimates of effect sizes (e.g. Cohen's d, Pearson's r), indicating how they were calculated

Our web collection on statistics for biologists contains articles on many of the points above.

Software and code

Policy information about availability of computer code

Data collection	custom software was used for data acquisition (nanomotion signal) that works in concert with the nanomotion device (Phenotech)
Data analysis	Microsoft Excel, GraphPad Prism 10, Python 3.9.2

For manuscripts utilizing custom algorithms or software that are central to the research but not yet described in published literature, software must be made available to editors and reviewers. We strongly encourage code deposition in a community repository (e.g. GitHub). See the Nature Portfolio guidelines for submitting code & software for further information.

Data

Policy information about availability of data

All manuscripts must include a data availability statement. This statement should provide the following information, where applicable:

- Accession codes, unique identifiers, or web links for publicly available datasets
- A description of any restrictions on data availability
- For clinical datasets or third party data, please ensure that the statement adheres to our policy

Data associated with figures is available in the Source data file and in the Supplementary data files. A comprehensive list of bacterial strains is provided in Supplementary Data File 1. For the development and testing of classification models, detailed information about recordings, their connection to samples, and associated strains can be found in Supplementary Data File 2. The description of each SP used in these models can be found in the Supplementary Information file.

April 2023

Corresponding SP values and scores can be found in Supplementary Data File 2. The clinical study protocol for Phenotech-1 is summarized at https://clinicaltrials.gov/study/NCT05613322.

Research involving human participants, their data, or biological material

Policy information about studies with human participants or human data. See also policy information about sex, gender (identity/presentation), and sexual orientation and race, ethnicity and racism.

Reporting on sex and gender	No data on sex and gender was collected. A sex and gender-based analysis would have no impact on the generalizability and translation of the findings.
Reporting on race, ethnicity, or other socially relevant groupings	No data on race and ethnicity was collected.
Population characteristics	Participants > 18 years old (all clinical and pre-clinical experiments), healthy (pre-clinical experiments only) or with positive blood cultures for either E. coli or K. pneumoniae (for clinical study only).
Recruitment	Blood from healthy donors used to generate spiked cultures was supplied by a blood donation center in Switzerland. A consent form was signed by each donor. All bacterial isolates used in the pre-clinical experiments derived from hospital collections or biobanks like IHMA, ATCC or the Swiss National Reference Center for Antibiotic Resistance. All bacterial isolates used in the clinical study are collected at each investigational site according to the eligibility criteria specified in the study protocol. In Lausanne and Innsbruck, participants had signed a general consent agreeing to the use of their residual biological material. In Madrid, consent for participation was not required for this study in accordance with institutional requirements.
Ethics oversight	An ethics approval for the pre-clinical experiments was not required as anonymized biological material was used. The clinical study protocol was approved by the Ethics Committee for Investigation with Medicinal Products (CEIm) in Madrid (ID 239/22), the Cantonal Commission for Ethics in Research on Human Beings (CER-VD) in Lausanne (ID 2022-02085), and the Ethics Committee of the Medical University of Innsbruck in Innsbruck (ID 1271/2022).

Note that full information on the approval of the study protocol must also be provided in the manuscript.

Field-specific reporting

Please select the one below that is the best fit for your research. If you are not sure, read the appropriate sections before making your selection.

☒ Life sciences ☐ Behavioural & social sciences ☐ Ecological, evolutionary & environmental sciences

For a reference copy of the document with all sections, see nature.com/documents/nr-reporting-summary-flat.pdf

Life sciences study design

All studies must disclose on these points even when the disclosure is negative.

Sample size	The aim of this study was to develop classification models that reliably delineate resistant and susceptible bacteria to antibiotics. For the sample size, the main point of reference was the diversity of bacterial strains regarding their minimal inhibitory concentration to the antibiotic (MIC), i.e., we tried to cover the entire MIC spectrum as well as possible. Mathematically, the lower limit of the number of experiments is given by the number of signal parameters used for a model. The number of signal parameters should not exceed the square root of the number of experiments. For instance, a model using 4 signal parameters should have been trained and cross-validated on 16 experiments. All our models exceed this by far.
Data exclusions	No data were excluded from the analysis.
Replication	In Figures 3, 5 and 6 the performance calculation is based on single recordings for which a score was calculated. Each recording is depicted as a data point representing a biological replicate originating from a different PBC. Performance calculation in Figure 4 is based on the median of the scores calculated for each technical replicate originating from the same PBC. Experiments were done in technical replicates - usually triplicate measurements. All experiments and information, including information about the number of strains or clinical isolates, samples and recordings, can also be found in the supplementary data file 2. Each nanomotion recording/experiment was given a unique identifier number. Technical replicates of one sample are given a sample ID. Each strain a unique strain ID and a number of strains were measured in different samples (different spiked blood cultures) with the same antibiotic (biological replicates).
Randomization	Not applicable. Number and type of experiments were defined to cover the entire MIC spectrum and were dependent on the signal parameters used for the model.
Blinding	Not applicable. Assessment of the isolate type and data is needed during collection and analysis.

Reporting for specific materials, systems and methods

We require information from authors about some types of materials, experimental systems and methods used in many studies. Here, indicate whether each material, system or method listed is relevant to your study. If you are not sure if a list item applies to your research, read the appropriate section before selecting a response.

Materials & experimental systems

n/a	Involved in the study
☒	☐ Antibodies
☒	☐ Eukaryotic cell lines
☒	☐ Palaeontology and archaeology
☒	☐ Animals and other organisms
☐	☒ Clinical data
☒	☐ Dual use research of concern
☒	☐ Plants

Methods

n/a	Involved in the study
☒	☐ ChIP-seq
☒	☐ Flow cytometry
☒	☐ MRI-based neuroimaging

Clinical data

Policy information about clinical studies

All manuscripts should comply with the ICMJE guidelines for publication of clinical research and a completed CONSORT checklist must be included with all submissions.

Clinical trial registration	NCT05613322
Study protocol	A summary of the protocol can be found at clinicaltrials.gov. The full protocol can be made available upon termination of the study.
Data collection	Data collection takes place at the following investigational sites: Lausanne University Hospital (Lausanne, Switzerland), University Hospital Ramón y Cajal (Madrid, Spain), and Medical University of Innsbruck (Innsbruck, Austria). At each site, remnants of positive blood cultures are obtained by the study team from the hospital diagnostic laboratory. Bacteria are isolated and used to perform an antibiotic susceptibility test with the Phenotech device and the reference method. The results of the tests are collected in the study database. Recruitment and data collection started in January 2023 and are expected to be completed by mid-2024.
Outcomes	Primary and secondary outcomes were defined and calculated according to ISO 20776-2 (2021) and EUCAST AST guidelines. Time to result was calculated as mean hours from the start of the antibiotic susceptibility test to the generation of the result in form of a time stamped report.
